# Fast Methods for Drug Approval: Research Perspectives for Pandemic Preparedness

**DOI:** 10.3390/ijerph20032404

**Published:** 2023-01-29

**Authors:** Ahmad Yaman Abdin, Francesco De Pretis, Jürgen Landes

**Affiliations:** 1 Division of Bioorganic Chemistry, School of Pharmacy, Saarland University, D-66123 Saarbrucken, Germany; 2 Department of Communication and Economics, University of Modena and Reggio Emilia, 42121 Reggio Emilia, Italy; 3 VTT Technical Research Centre of Finland Ltd., 70210 Kuopio, Finland; 4 Department of Philosophy “Piero Martinetti”, University of Milan, 20122 Milan, Italy

**Keywords:** computer-generated evidence, decision making, drug approval, evidence synthesis, pandemic, pandemic preparedness, real world evidence, risk attitudes

## Abstract

Public heath emergencies such as the outbreak of novel infectious diseases represent a major challenge for drug regulatory bodies, practitioners, and scientific communities. In such critical situations drug regulators and public health practitioners base their decisions on evidence generated and synthesised by scientists. The urgency and novelty of the situation create high levels of uncertainty concerning the safety and effectiveness of drugs. One key tool to mitigate such emergencies is pandemic preparedness. There seems to be, however, a lack of scholarly work on methodology for assessments of new or existing drugs during a pandemic. Issues related to risk attitudes, evidence production and evidence synthesis for drug approval require closer attention. This manuscript, therefore, engages in a conceptual analysis of relevant issues of drug assessment during a pandemic. To this end, we rely in our analysis on recent discussions in the philosophy of science and the philosophy of medicine. Important unanswered foundational questions are identified and possible ways to answer them are considered. Similar problems often have similar solutions, hence studying similar situations can provide important clues. We consider drug assessments of orphan drugs and drug assessments during endemics as similar to drug assessment during a pandemic. Furthermore, other scientific fields which cannot carry out controlled experiments may guide the methodology to draw defeasible causal inferences from imperfect data. Future contributions on methodologies for addressing the issues raised here will indeed have great potential to improve pandemic preparedness.

## 1. Introduction

Public heath emergencies, such as the outbreak of novel infectious diseases, are a major challenge for drug regulatory bodies, practitioners, and scientific communities. The COVID-19 pandemic, for example, has catapulted pandemic preparedness onto the public stage [1] and spurred a great number of academic works [2,3,4,5]. Pandemic-related topics have been so hot that even the normally slow-moving field of philosophy has shown interest in conferences [6], published dedicated Special Issues [7,8,9,10], and stand-alone papers [11]. Much has been said, argued, and learned. Despite a flurry of activities in public and academic domains concerning pandemic preparedness, little has been said about how to make decisions on whether to implement and/or approve medical interventions, such as drugs, to combat the next pandemic(s).

Drug approval and authorisation are essential mechanisms for regulating and ensuring the safety and effectiveness of pharmaceutical products. Under normal conditions and circumstances, the process of drug development and approval is lengthy and expensive, the success rate is low and subsequent clinical implementation is slow [12,13].

Effectively combating pandemics, however, crucially requires rapid decision making [14]. Yet, uncertainty is inherent in such urgent circumstances due to time pressure which renders adequately powered and sufficiently long-lasting randomised clinical trials (next to) impossible to conduct [15,16]. In the lack or absence of randomised evidence, decisions must be, at least partially, based on non-randomised evidence, sometimes referred to as Real World Evidence (RWE). RWE is a clinically relevant type of evidence for drawing inferences. It is characterised by the different observational data obtained outside the context of Randomised Controlled Trials (RCTs).

A conceptual analysis of decision making in the context of drug approval during pandemics can identify at least three separate, but intimately connected, issues. Firstly, which risk attitudes ought we to have? This issue concerns the approach to managing risk in drug approval decision making under substantial uncertainties. Secondly, which methodology for evidence production ought we to use? When adequate RCTs are not feasible, the focus shifts to study designs which will generate the best possible evidence to support the safety and effectiveness of a drug. Thirdly, how should we synthesise the available evidence? Here, we turn our attention to methods for reducing bias and aggregating the available evidence to support such decision making. Progress on these undeniably hard questions concerning our methodology for approving drugs to protect, treat, or cure patients during pandemics can indeed contribute to an improved pandemic preparedness and ultimately better public health outcomes. It should be noted that the term *pandemic preparedness* is often employed to only refer to human and physical resources. We here use a much wider sense including methodologies for uncertain inferences in public health decision making within the meaning of the term, cf. [17].

Therefore, the rest of the paper addresses these three points and considers the means to answer them. It is outside the scope of this article to resolve these issues/questions, our purposes are conceptual: (I) to clearly lay out relevant questions, (II) to present challenges for answering these questions, and (III) to point towards means for addressing such challenges to eventually provide solutions and, hence, increase pandemic preparedness. Our conceptual analysis is built upon the recent developments in both general philosophy of science, philosophy of medicine, and risk assessment theory.

Section 2 discusses the determination of rational risk attitudes. Section 3 elaborates on appropriate means for evidence production and Section 4 investigates the synthesis of available evidence. Each of these three sections is in turn divided into four parts. The first part is dedicated to posing pertinent questions (Section 2.1, Section 3.1 and Section 4.1), the second studies relevant concepts (Section 2.2, Section 3.2 and Section 4.2), the third points to some strategies for obtaining answers (Section 2.3, Section 3.3 and Section 4.3), and the fourth part exemplifies some lessons learned from fighting COVID-19 (Section 2.4, Section 3.4 and Section 4.4). Section 5 lays out our tentative conclusions.

## 2. Risk Attitudes

One important aspect of decision making is risk and all aspects of its management. Our risk attitudes towards a decision are influenced by—among other factors—the uncertainty related to available choices (courses of action) and their expected possible outcomes. Classical decision theory distinguishes three types of risk attitudes. A risk neutral attitude is characterised by making the choice that maximises expected utility regardless of the uncertainties of outcome. Risk aversion is an attitude characterised by trading off expected utility for a reduction in uncertainty; it is to be on the safe-side of things. On the contrary, risk seeking attitudes trade off expected utility for the possibility of greater returns and emphasise the possibility of better than expected outcomes.

As we set out next, risk attitudes concerning drug assessment during a pandemic are effected by a number of further risk attitudes.

### 2.1. A Set of Pandemic Risk Attitudes

We now list a number of pertinent risk attitudes for drug approval during a public health emergency. Due to the inherent large uncertainties risk attitudes towards uncertainty and the reduction of uncertainty play a large role in our analysis. We illustrate these risk attitudes as answers to the questions we pose and by pointing to examples during the COVID-19 pandemic. Relevant concepts are analysed in more detail in Section 2.2.

(1) How to judge large uncertainties about the magnitude of beneficial effects when authorising an intervention such as the use of a drug to prevent, treat, or cure a novel disease? The inherent uncertainty about the effectiveness and safety of Hydroxychloroquine to treat COVID-19 patients, for example, led to a careful approach in its consideration as a potential cure [18,19].

(2) How to judge large uncertainties about the magnitude of adverse reactions, in terms of severity and frequency, when authorising an intervention such as the use of a drug to prevent, treat, or cure a novel disease? The implication of the Oxford–Astra Zeneca vaccine in blood clotting, for example, led regulators also to adopt a careful approach in its application [20].

(3) How to trade off-risks (adverse reactions) of an intervention with its benefits? Again, the application of the Oxford–Astra Zeneca vaccine was suspended after reports of blood clots [20]. However, different countries have reacted at different times, which could be caused by their different risk attitudes or by differing speeds of decision making processes [16].

(4) How to regulate the drug market? Currently, the European Medicines Agency (EMA) and the Food and Drug Administration (FDA) control the market circulation of vaccines in the EU and the US, respectively. Only after the vaccines were approved by these drug regulators did they become available to the public [21].

(5) How to produce reliable evidence to reduce uncertainties about the safety and effectiveness of drugs? During the COVID-19 pandemic, animal experimentation was widely conducted [22]. By contrast, human challenge trials were very rare [23].

(6) How to distribute available scarce medical resources? In many countries around the world, critical workers and vulnerable patients were allocated the COVID-19 vaccines prior to the general public [24]. The number of ventilators was at times and places insufficient to treat all the patients [25].

(7) How to prioritise scarce research funds? Public and private research funds were both devoted to research therapies and preventive measures such as vaccines [26].

(8) How to mandate health interventions? In some countries, several vaccines are mandated for sub-populations (often health workers) [27].

This list is neither complete nor exhaustive. We acknowledge that every pandemic will be different in terms of public opinion and medical and public health challenges as well as the pathogens. Nevertheless, we strongly believe that pandemic preparedness can be increased by having clearer responses to these questions *prior*to the next pandemic(s) occur(s).

### 2.2. Concepts of Risk Attitudes

There are a number of distinct concepts involved in understanding and approaching risk attitudes. We now delineate such concepts regulators, scientists, doctors, patients, and other stake holders may hold in more detail.

(1) Benefit Assessments: Benefit assessments are—under normal circumstances—based on a well-stocked arsenal of statistical techniques that facilitate the quantification of expected benefits. Moreover, uncertainties can also be statistically quantified with respect to the expected average benefits and expected variance. Such issues are constantly discussed in the context of drug approval. However, if the body of available evidence during a pandemic does not permit a reliable quantification of an expected variance nor an expected average benefit, how should one react to the inherent uncertainties?

(2) Safety Assessments: Unlike benefit assessments, safety assessments need to track rare events in which medical interventions can led to dire, sometimes even fatal, consequences. The rarity of these events and the fact that randomised clinical trials are often small—in terms of patient numbers and duration—necessitates that safety assessments regularly invoke evidence from non-randomised trials [28,29,30,31]. As a result, narrative evidence reviews incorporating RWE appear regularly in safety assessments, while methodology for safety assessments based on non-randomised evidence is currently in use; a pronounced lack of randomised evidence will increase the uncertainty about the effect size of the expected adverse reaction, its variance, and patient groups with greater risks of suffering adverse reactions. How should one react to the inherent uncertainties, which are already hard to quantify under normal circumstances?

(3) Risk Benefit Trade-Off: Many decision theorists hold that the “normatively” correct way of trading off positive against negative consequences is to act in the way that maximises expected utility, i.e., avert risk [32,33,34]. This decision procedure trades off benefits with losses at the rate of 1 to 1. In the context of drug approval, it is thus at odds with the medical principle of primum non nocere: cause no harm! Clinicians and public health officials are well-versed in such trade-offs under normal circumstances. Given significant uncertainties discussed above under 1 and 2, how should the expected benefits be traded off against the expected adverse reactions?

(4) Drug Regulation: the EMA in the EU and the FDA in the USA regulate market entry and circulation of medicines restricting patients’ access to products deemed by these organisations(!) to be safe and efficacious. Patient groups and drug manufacturers have advocated for patients, with the guidance of their physicians, to have the autonomy to choose their own medications, as they are the ones who experience the effects of the drugs [35,36,37,38]. When there is little time and less (good) evidence than normal, should drug regulators provide patients with more freedom to choose?

(5) Ethics of Evidence Production: Human participants in randomised trials have normally consented to their participation while patients in observational studies are normally not intervened on by study designers. During a pandemic, the challenge arises to quickly generate large data sets. Human challenge trials, in which healthy volunteers are deliberately exposed to pathogens, can provide evidence much faster but do so at a significant risk to the volunteers’ healths [39,40]. Animal experimentation is a cheap way to quickly conduct randomised trials and inevitably carries the significant risk of animal suffering [41]. Animal rights activists and some ethicists argue for “replacement, reduction, refinement” through (i) in vitro and in silico rather than animal experimentation, (ii) fewer experiments with animals, and (iii) minimising pain and distress [42]. In view of large-scale human suffering, we might accept to cause more harm to animals in order to benefit human patients. How should ethical committees/requirements deal with human challenge trials and/or animal experimentation during a pandemic?

(6) Resource Distribution: Drugs regulated by EMA and/or the FDA tend to be available in the necessary quantities in the EU and the USA. They also tend to have reasonably-well understood risk–benefit profiles. During a pandemic, drugs may be scarce and poorly understood, which may cause a need to prioritise their administration to subgroups of the population. The prioritisation of vulnerable subgroups and essential (health) workers has been discussed and implemented [43]. However, if the intervention causes more harm than good, then critical (health) work might not be completed and the already vulnerable have to deal with further health complications. How should scarce medicines with relatively poorly understood risk–benefit profiles be prioritised?

Further hard ethical questions arise concerning the distribution of scarce available resources; we now briefly mention some. (a) Should all patients be treated equally or should patients with a greater life expectancy and/or quality of life receive preferential treatment? In essence, should scarce resources be allocated to children rather than old-age pensioners—all other things being equal? Further complicating this question, are the different risk profiles and prospects for recovery for different age groups. Indeed, age appears to be an important covariate of effectiveness and safety of vaccines [44]. Furthermore, after vaccinating (most of) the population twice, should further vaccine doses be used as booster shots or should some be donated to less fortunate countries with much lower vaccination rates attributed to availability [45]?

(7) Distribution of research funds: Similar to the other risk attitudes mentioned here, different actors had different attitudes. For example, the World Health Organization funded the solidarity trials in order to assess the viability of existing drugs for fighting the pandemic [19]. Existing drugs are potentially cheaper to manufacture and distribute, have a better understood risk profile, are already in stock, and, crucially, do not require a long and expensive discovery phase. The lower cost causes them to be particularly interesting for less wealthy countries and patients. In line with this assumption, most of the funded trials on ClinicalTrials.gov were investigating treatments [26] rather than vaccines.

(8) Voluntariness of Health Interventions: Unlike regular illnesses that threaten relatively small subgroups, pandemics have the potential to wipe out entire populations and destroy much of the society. Individuals hence have a greater responsibility towards fighting pandemics. Given this greater responsibility, should (public) health interventions be mandated more often? Since patients with symptoms are more likely to spread pathogens than patients without symptoms, mandatory health interventions on patients with symptoms seem ethically less objectionable.

### 2.3. Determining Risk Attitudes

Next, we present two main strategies to determine risk attitudes.

Firstly, one can extrapolate risk attitudes from similar situations either in terms of past pandemics or current public health crises to inform debates about rational risk attitudes during future pandemics. Past pandemics provide us with ample evidence of risk attitudes of decision makers (Section 2.1). With the benefit of hindsight, we may be able to determine whether these risk attitudes led to desired outcomes and how they could thus guide us in future times of crisis. Furthermore, currently, hyperendemic diseases such as malaria [46], HIV/AIDS, and hepatitis [47] in Ghana force clinicians and public health officials to make hard choices. Their risk attitudes can either be studied from their actions or elicited directly. Informing our thinking with risk attitudes of health professions of an ongoing public health crisis can provide us with vital insight to steer our actions in future crises. Johnson and Vindrola-Padros found that, in past outbreaks, especially outbreaks of Ebola, quarantine decisions were not easy and could also generate undesirable effects, even precipitating community resistance to viral control [48].

Secondly, one can apply theoretical arguments to enrich such debates. Normative ethical theories constrain our actions aiming to ensure that we do the right thing. Relevant works concern the degree of market control to be exercised by drug licensing agencies [49], trade-offs between risks and benefits [50], how to react to unknown risks [51], animal welfare [41], and human challenge trials [39]. For example, utilitarianism holds that morally right actions produce the most good, where the goods produced for individuals count equally. As a consequence, the overall good can be computed as a *non-weighted sum* of individual goods [52]. Utilitarianism entails that the primum non nocere dictum can be violated in cases in which the overall goods of the population outweigh the negative consequences suffered by some; e.g, rare ADRs caused by an effective vaccine mean that vaccinating the population is not only ethically permissible but ethically mandatory.

### 2.4. Lessons Learned concerning Risk Attitudes

We shall now attempt to tentatively outline some lessons learned from fighting the COVID-19 pandemic. The communication of risks is an important factor in fighting pandemics [53,54]; the point is also echoed by EMA [16]. Lancaster et al. [55] have highlighted the various perspectives on the connection between evidence and decision making. They argue that models for rapid assessment and outbreak science have demonstrated alternative ways of creating evidence during emergencies. Additionally, they point out that during crises, particularly the outbreak of a new infectious disease, the generation and synthesis of evidence, as well as decision making and public health actions, occur simultaneously. They suggest adopting a reflexive and adaptable approach that involves ongoing communication between decision makers and affected communities.

## 3. Evidence Production

There are a number of study design characteristics that allow for reliable inferences under normal circumstances. However, when studies with such characteristics are not available, we instead have to rely on expert opinion, mechanistic modelling and observational evidence. In what follows, we begin by posing the question of how to design a good study during a pandemic. We then analyse the relevant characteristics and discuss how to improve learning from imperfect data.

### 3.1. Starting Point

Given our starting premise for a need of rapid decision making and rapid evidence production, there will likely not be sufficient time to run adequately powered randomised studies long enough. Apart from lacking biological and epidemiological understandings of the pathogen, early on in a pandemic patients participating in vaccine trials might not be exposed to this pathogen often enough to reliably assess the effectiveness of medical interventions [56]. Similarly, trials of therapies will plausibly struggle to recruit sufficient numbers of patients [57]. In order to make the best possible decisions in such a challenging situation, we would prefer to have the best possible evidence available. How can we craft study designs that will provide maximally informative and reliable evidence?

### 3.2. Concepts of Study Design

Determining the best study design a priori without making assumptions about the particular situation is not a feasible undertaking. We shall instead consider the properties of study design that have been thought to be conducive to the production of reliable causal inferences.

(1) The duration of the observation of patients is a part of the design that can be controlled. In particular, the duration of the observation is key for learning about any possible drug-related complications or adverse reactions [58,59,60]. Although a long duration is a key ingredient for learning about the unsafety of a drug, time is of the essence and is not available in abundance during a pandemic.

(2) The number of patients to be enrolled in trials is also under our control. Large sample sizes are one of the statistical main features to produce reliable causal inferences. Again, our premise stands in the way of achieving this, since we assume that time pressure will not permit the running of large trials due to a combination of: lack of volunteers, lack of drug doses, lack of time to prepare clinical trials, and low chances of patients coming in contact with the pathogen. Furthermore, a large sample size only provides us with a reason to believe that the measured effect size is not due to random noise (precision) but no guarantee that the measured effect size is accurate: precision is no guarantee for accuracy [61].

(3) Conflicts of interest are known to cause biased study design and interpretation [62,63]. Some have gone as far as demanding nationalisation of drug development, testing, and monitoring [49]. In the case of a pandemic, excluding the experts developing the drugs from monitoring and interpreting studies comes with the risk of depriving us of the only available intimate knowledge available. We hence think that the less drastic option of excluding drug developers from drug monitoring in an effort to reduce bias is a double-edged sword in the current market economy governing drug development.

(4) The adjustments for covariates at the design and analysis stage are another option to reduce bias. Adjustment techniques are already the current best practise in randomised trials and observational studies given that certain conditions are satisfied [64,65]. A continuation of this best practise approach appears to be the sensible course of action during a pandemic.

(5) Evidential variety was long held to be, ceteris paribus, desirable. For example, it was strongly believed that, when comparing (A) conducting two studies with two different but equally probative study designs that show the same result with (B) conducting two different studies with the same study design that show the same result as the former studies, option A would allow for stronger conclusions. This long-held belief is surprisingly not universally true and fails at times [66,67,68,69,70].

(6) Experimentation on surrogate models (animals, in vivo, in vitro, and in silico) can be conducted on much larger scales for a much lower cost and, thus, has features that cause it to be well-suited for current purposes. However, in order to reliably infer from the indirect evidence obtained from such models about humans one needs to ensure that the experimental results are also externally valid. Direct evidence is, by contrast, obtained from patients within a given population of interest. Inferring causal relationships about a particular patient in the population of interest from observed/estimated frequencies in the population is a related issue [71].

The choice of a reference class population is another thorny issue: choosing a large reference class population can result in a heterogeneous class of patients that do not all closely resemble the patient of interest, choosing a narrow reference class population instead can lead to few observations causing reliable frequency estimations to be challenging [72]. There is hence a trade-off between the data quality and data quantity to be struck.

External validity is often sought via analogical reasoning [73,74,75] by demonstrating that the model and humans are (sufficiently) similar in the relevant respects. However, the relevant respects are defined as those that vouch for the (sufficient) similarity between the model and humans. This circle of analogy referring to ‘the relevant respects ensuring that the analogy holds’ is clearly vicious and known as the *circle of extrapolation* [76]. There does not seem to be a way to break this vicious circle casting an unfavourable light on the evidence obtained from experimentation on surrogate models. Nevertheless, the lure of causing analogical reasoning to work continues to attract attention [77,78].

(7) The blinding of patients and medical staff is a key part of RCT methodology widely believed to increase the reliability of the causal signals inferred from trials. The blinding of statisticians carrying out data analysis can be implemented in randomised trials and observational studies alike. While the blinding of statisticians during the data analysis is often implemented and thought to increase the reliability of causal inferences, there are however a number of (mutually incompatible) ways to implement the blinding of statisticians; determining the appropriate procedure is not always trivial [79].

(8) Large measured effect sizes can be one of the properties (of meta-analyses) of studies which most convince us that a drug is efficacious. However, a measured effect size is not a property of the *design* of the study and thus not directly influenced by (near) optimal study design. Furthermore, an uncannily large effect size might instead be a sign of a bias swamping the signal and thus, instead, indicate poor design [80].

### 3.3. Life without Perfect Data

We now turn to possible ways to infer from imperfect data. We present two main strategies: firstly, we can learn from other sciences engaging in extrapolation. Secondly, we turn to the assessment of orphan drugs.

(1) Other sciences have to deal with imperfect data on a regular basis. Consider for example astrophysics, where we cannot, for obvious reasons, directly intervene on black holes, but we can experiment with table-top set ups (dumb holes) that are analogous to black holes in some sense [81]. Nutrition scientists are very interested in learning about the causal effects of our diets on our health. Since causal effects are normally small and require years to manifest, randomisation is not an option [82]. Despite these challenges, we now have a clear understanding of what constitutes a healthy diet [83]. In macroeconomics, one would very much like to understand the causal effect of policies (minimum wage, free universal health care, and taxation rates) on economic outcomes of interest (GDP, median household income, and poverty). However, we cannot randomise a sufficiently large number of countries (which are sufficiently homogeneous), intervene via policy changes, and calculate a causal effect. Economists are drawing on two main strands to make predictions: (1) observational data that may be biased and (2) economic theory, which might not apply (or be mis-calibrated) to the problem at hand [84]. Despite regularly dealing with imperfect data, nutrition science and macroeconomics are thriving scientific disciplines. Studying their causal inference procedures may improve our thinking about combating pandemics.

(2) Orphan drugs treat, by definition, rare medical conditions [85]. Due to their intended application, the effectiveness and possible adverse reactions of an orphan drug are by definition poorly understood at the time of drug approval. The approval of orphan drugs is hence similar to drug approval in a public health emergency. The lessons learned from assessing orphan drugs might hence be fruitfully applied to drug assessments during a pandemic.

### 3.4. Lessons Learned concerning Evidence Production

The presence of a natural control group is a widely appreciated key piece of RCT methodology. It facilitates the computation of effectiveness in RCTs compared to the control group of patients, which can be subject to the placebo effect. The nocebo effect by contrast concerns patient-reported *un*favourable health outcomes after exposure to harmless stimuli. The frequency and severity of nocebo reporting are known to depend on risk communication (see Section 2.4) and have been studied for COVID-19 [86,87]. The observed nocebo effects can be used to predict the frequency and severity of noceobo effects in future pandemics.

In order to increase the sample size of studies/trials, (i) inclusion/exclusion criteria can be formulated to exclude few patients, (ii) patients can actively be recruited, and (iii) the number of patients may not be pre-determined as in the case of the solidarity trial [88]. While such methodology can surely help increase the size of future study/trial populations, we are doubtful that such measures will suffice on their own to solve the problem of small samples.

Adopting a wider perspective, arguments have been put forward against the exclusion of social and contextual factors from evidential reasoning in data science. Instead, a “multidisciplinary, reflexive, socially attuned, and engaged approach to research can go a long way toward fostering robust, reliable, and responsible outcomes, despite requiring more time and resources to set up” [89]. Furthermore, there is a demand to be more transparent with evidence judgements (which data to collect, how to measure quantities of interest, which models to use, etc.) and to discuss these evidence judgements openly rather than behind closed doors or sweeping them under the carpet [90].

## 4. Evidence Synthesis

We now turn to the challenge of synthesising the available evidence.

For special circumstances of major public health interest, regulatory bodies such as the EMA and the FDA developed strategies to expedite the drug approval process. Such strategies included the accelerated evaluation and assessment of marketing applications and authorisation for the emergency use of medical products [91,92]. These strategies are broad in nature and rather unspecific, e.g., requiring a positive benefit–risk trade-off and a case-by-case analysis. Highly successful COVID-19 vaccines were approved using these strategies [93]. Our starting point is a need for improving these strategies for evidence synthesis to better manage future pandemics.

### 4.1. Synthesis of the Available Evidence

As we laid out in the previous section, decision making will be based on a body of evidence containing RWE (real world evidence) in the form of observational studies at the population level, animal studies, bench research, and/or in silico clinical trials. The challenge arises to make the best possible use of all this information. How would we best synthesise the body of available evidence?

### 4.2. Concepts of Real World Evidence Synthesis

Ideally, one would like to have a method to de-bias the available evidence to obtain reliable information about causal relationships. Unfortunately, no such promising method has been suggested. Cleaning bias from data is a pipe-dream, yet.

Our best developed concepts and formal methods for aggregating data are meta-analyses built on statistical principles. Meta-analyses struggle with incorporating data from studies with different designs (randomised, case-controlled, longitudinal, and cross-sectional). In order to also include data from experimentation on surrogate models more general but less formal methodology of evidence synthesis is required. Less formal methods of evidence synthesis are already used by drug licensing agencies. Such methods are, in particular, required to also include case reports that play a key role in pharmacovigilance [28,30,94,95].

Given the need for evidence synthesis methods for pharmacovigilance that can deal with bodies of evidence containing biased evidence of a variety of sorts (randomised, case-controlled, longitudinal, cross-sectional, animal (toxicology) studies, case reports, etc.), It is not surprising that a great number of approaches have been developed addressing respectively the quality/bias of studies or the synthesis of evidence.

Rather than suffering from a lack of concepts and methodology [96], the problem instead is that there are too many methods available and we do not have a clear understanding of which method to apply in each case. This problem of an excess of methodology has been observed for quality assessment tools [97], meta-analyses [98], and evidence synthesis [99]; see also [100,101,102,103,104].

### 4.3. The Concept Uncertainty in Evidence Synthesis of Real World Evidence

While there is significant philosophical interest in evidence synthesis from deep foundational perspectives [105,106], it is not clear how to deal with significant uncertainties. As we argued in Section 4.2, purely statistical methods are ill-suited for evidence synthesis for drug assessments during a pandemic. We now discuss further concepts of uncertainty that may be useful for uncertain causal inference to support drug approval during a pandemic.

The most popular approach to uncertainty in the philosophy of science and the philosophy of medicine is *Bayesian epistemology* [107,108]; for a short introduction, see Appendix A4 in [109]. Similar to Bayesian statistics, uncertain inferences in Bayesian epistemology are based on (i) prior probabilities capturing the background knowledge and (ii) updating procedures (Bayesian or Jeffrey) to model the acquisition of evidence [110]. Unlike Bayesian statistics, Bayesian epistemology defines probabilities over all the variables one is interested in. It is built upon the fact that accumulating evidence overrides prior beliefs and the posterior beliefs based on truth-conducive evidence converging to the truth [111]. This puts one in a position to conduct evidence synthesis based on all the available evidence [112,113,114,115] by calculating the probabilities of effectiveness and safety of drugs. Unfortunately, Bayesian evidence synthesis requires the specification of a large number of relevant probabilities, which cannot be defined in an objectively correct way and must be fixed subjectively [116]. The debate on the seriousness of this subjectivity has been raging for many decades [117,118,119,120]. Let it suffice to say here that an end of this debate is not in sight. In our view, a rational choice affecting millions of lives ought to not hinge on the subjective choice of prior probabilities.

Some authors maintain that the choice of prior probabilities is not as subjective as alleged; while this may be so, it moves Bayesian epistemology closer to the competing, but less popular, *objective Bayesian epistemology*. This epistemology posits an objective relation between evidence and prior probabilities and offers a more powerful machinery for updating beliefs capable of handling arbitrary probabilistic information [121,122]. This objective Bayesian approach requires a canonical model to assign probabilities to the propositions of interest. This epistemology applies the principle of indifference to set prior probabilities and then appeals to an inferential principle of entropy maximisation to update probabilities in the light of new evidence. Choosing a different model yields, in general, different probabilities to the same proposition of interest [123]. Unfortunately, it is not at all clear what the canonical model is in practical applications. Rather than suffering from a subjectivity of probabilities, this epistemology requires a non-objective choice of a canonical model.

A different popular approach to uncertainties is the framework of *imprecise probabilities*, which uses *sets of probabilities* to model our ignorance, e.g., the probability of a fever being caused will be between 15% and 25%. This epistemology is well-suited to capture the significant uncertainties in our decision problem [124,125,126]. The main issue with this epistemology is that there is not one but many plausible ways to use sets of probabilities for decision making [127]. Some of these ways will not recommend an action at all on the grounds of insufficient evidence; while not making a choice is an option in many instances, a choice must be made in our decision problem. Unfortunately, no clear choice procedure is emerging for this epistemology.

A number of further approaches exists, which we now briefly discuss. *Qualitative decision theory* [128] aims to improve decision making by not employing numbers for quantification, which is in a spirit related to the idea behind the development of imprecise probabilities. Similarly, there is a lack of agreement on the appropriate choice procedure. Furthermore, the quantification of effect sizes is a pillar of medical decision making; ignoring the magnitude of effect sizes seems inadvisable. *Ranking functions* capture uncertainty by assigning outcomes a unique rank in the set {0,1,2,3,…,∞} [129]. Again, it is (still) unclear, how to make rational decisions using ranking functions and no methodology has been developed for transforming medical evidence into a ranking function. *Severe testing* is designed to reason about statistical hypotheses by putting them to severe tests [130]. Finally, *choice under complete uncertainty* refers to decisions made in the absence of quantitative utilities [131,132], while *ambiguity* refers to choices without knowledge of the underlying state space [133].

Despite—or because of—these foundational issues, *machine learning and artificial intelligence more generally* for evidence synthesis are becoming increasingly popular [134,135,136,137,138,139,140,141]. Philosophical enquiries on how computer simulations methods can help with handling evidence have been discussed from a foundational point of view [142,143], theoretical limits [144], and added value [145].

### 4.4. Lessons Learned concerning Evidence Synthesis

Unfortunately, we are not aware of significant advances in evidence synthesis of real world evidence due to COVID-19 research. An outline of some of the challenges ahead has recently been provided by four EMA representatives [16]. In [55], Lancaster et al. went as far as to “suggest that the unprecedented challenges of COVID-19 do not simply require us to speed-up existing evidence-based approaches, but rather necessitate different ways of thinking about how a more adaptive evidence making cannot only contribute to addressing uncertainties, but do so in a manner responsive to an emergent and evolving situation”. They further that “this requires an openness to forms of evidence and expertise often absented from ‘business-as-usual’ evidence-based intervention.” A less drastic shift from EBM principles to EBM+ [146], which heavily emphasises mechanistic evidence, has also been suggested [147].

## 5. Conclusions

Approving new drugs is of crucial importance towards mitigating the multifaceted consequences of pandemics. In this paper, we discussed aspects of medical uncertain inference to increase pandemic preparedness. The stated long-term goal is to formulate a methodology for a streamlined and structured way to produce, synthesise, and use evidence for (public) health decision making that can contribute to the fight against future pandemics.

We pointed to the lack of (public) debate on ethical aspects and the absence of academic work on key methodological issues. The focus and prioritisation have been to flesh out the conceptual breadth and implications of hard problems. We followed up with discussions of arising challenges and possible ways to overcome them. The lists of issues and possible solution strategies we put forward, e.g., (i) turn to normative ethical theory and study endemics (Section 2.3) and (ii) learn from other disciplines (Section 3.3), are meant to indicate the complexity of the challenges ahead and gesture towards possible solutions. In particular, they are not meant to be complete. We think that adding further entries to these lists and improving on our thinking above is valuable future research. Useful insights can be gained from a number of areas such as medicine, public health, risk assessment theory, philosophy of science, philosophy of medicine, ethics, and sociology. In particular, learning from preparedness for other disasters seems to be a logical next step to take [148,149,150].

We only addressed some concepts pertaining to pandemic preparedness. Other relevant social aspects such as (i) knowledge and decision making being distributed among a number of individuals and institutions [151], (ii) the relevance of communication of expected health outcomes for reported health outcomes (placebo and nocebo) [48,87], (iii) how a society understands the concept of disease [152], (iv) impact on health workers [153], and (v) the uneven distribution of recovery (speeds) in societies [154] are discussed elsewhere.

Fighting a pandemic before it becomes a global public health crisis represents the best way to mitigate risks and prevent negative health outcomes. Interdisciplinary work and novel research methods will be of importance for achieving these aims.

## Data Availability

Not applicable.

## References

[1] Helmuth L. (2022). Introducing a Special Issue on How COVID Changed the World. Scientific American. https://www.scientificamerican.com/article/introducing-a-special-issue-on-how-covid-changed-the-world/.

[2] Pak A., Adegboye O.A., Adekunle A.I., Rahman K.M., McBryde E.S., Eisen D.P. (2020). Economic Consequences of the COVID-19 Outbreak: The Need for Epidemic Preparedness. Front. Public Health.

[3] Bashkin O., Otok R., Leighton L., Czabanowska K., Barach P., Davidovitch N., Dopelt K., Duplaga M., Emegwa L.O., MacLeod F. (2022). Emerging lessons from the COVID-19 pandemic about the decisive competencies needed for the public health workforce: A qualitative study. Front. Public Health.

[4] Gonzalez-Aquines A., Kowalska-Bobko I. (2022). Addressing health corruption during a public health crisis through anticipatory governance: Lessons from the COVID-19 pandemic. Front. Public Health.

[5] Miglietta A., de Waure C., Chronaki C., Wild C., Favaretti C., Timen A., Edelstein M., Petelos E. (2021). Health technology assessment applied to emergency preparedness: A new perspective. Int. J. Technol. Assess. Health Care.

[6] Institute for the Future of Knowledge at the University of Johannesburg, South Africa (2021). Philosophical Perspectives on COVID-19, 10–13 May 2021. Philosophy of Medicine. https://mms.philsci.org/Calendar/moreinfo_responsive.php?eventid=63376&org_id=PSA.

[7] Klonschinski A. (2020). Philosophie in der Pandemie? Einleitung: “Die Corona-Pandemie – Praktische Philosophie in Ausnahmesituationen”. Z. Fur Prakt. Philos..

[8] Boniolo G., Onaga L. (2021). Seeing clearly through COVID-19: Current and future questions for the history and philosophy of the life sciences. Hist. Philos. Life Sci..

[9] Leonelli S. (2022). Introduction: Biomedical knowledge in a time of COVID-19. Hist. Philos. Life Sci..

[10] dos Reis R.R., Pinto J.C.O., Nobre B., Lind A.G., Batista R.B. (2021). Thinking the Pandemic: Philosophical Perspectives. Rev. Port. De Filos..

[11] Aronson J.K., Auker-Howlett D., Ghiara V., Kelly M.P., Williamson J. (2020). The use of mechanistic reasoning in assessing coronavirus interventions. J. Eval. Clin. Pract..

[12] Scannell J.W., Blanckley A., Boldon H., Warrington B. (2012). Diagnosing the decline in pharmaceutical R&D efficiency. Nat. Rev. Drug Discov..

[13] Sun D., Gao W., Hu H., Zhou S. (2022). Why 90 percent of clinical drug development fails and how to improve it?. Acta Pharm. Sin. B.

[14] Eisman A.B., Kim B., Salloum R.G., Shuman C.J., Glasgow R.E. (2022). Advancing rapid adaptation for urgent public health crises: Using implementation science to facilitate effective and efficient responses. Front. Public Health.

[15] Glasgow R.E., Battaglia C., McCreight M., Ayele R.A., Rabin B.A. (2020). Making Implementation Science More Rapid: Use of the RE-AIM Framework for Mid-Course Adaptations Across Five Health Services Research Projects in the Veterans Health Administration. Front. Public Health.

[16] Cavaleri M., Sweeney F., Gonzalez-Quevedo R., Carr M. (2021). Shaping EU medicines regulation in the post COVID-19 era. Lancet Reg. Health-Eur..

[17] Rosário R., Fronteira I., Martins M.R.O., Augusto C., Silva M.J., Messer M., Martins S., Duarte A., Ramos N., Rathmann K. (2022). Infodemic Preparedness and COVID-19: Searching about Public Health and Social Measures Is Associated with Digital Health Literacy in University Students. Int. J. Environ. Res. Public Health.

[18] Graef E.R., Liew J.W., Putman M.S., Simard J.F., Sirotich E., Berenbaum F., Duarte-García A., Grainger R., Harrison C., Konig M.F. (2020). Festina lente: Hydroxychloroquine, COVID-19 and the role of the rheumatologist. Ann. Rheum. Dis..

[19] WHO Solidarity Trial Consortium (2021). Repurposed Antiviral Drugs for COVID-19 – Interim WHO Solidarity Trial Results. New Engl. J. Med..

[20] Wise J. (2021). COVID-19: European countries suspend use of Oxford-AstraZeneca vaccine after reports of blood clots. BMJ.

[21] European Medicines Agency (2020). EMA Recommends First COVID-19 Vaccine for Authorisation in the EU. Philosophy of Medicine. https://www.ema.europa.eu/en/news/ema-recommends-first-COVID-19-vaccine-authorisation-eu.

[22] Genzel L., Adan R., Berns A., van den Beucken J.J., Blokland A., Boddeke E.H., Bogers W.M., Bontrop R., Bulthuis R., Bousema T. (2020). How the COVID-19 pandemic highlights the necessity of animal research. Curr. Biol..

[23] Killingley B., Mann A.J., Kalinova M., Boyers A., Goonawardane N., Zhou J., Lindsell K., Hare S.S., Brown J., Frise R. (2022). Safety, tolerability and viral kinetics during SARS-CoV-2 human challenge in young adults. admissions and intensive care admissions from COVID-19. Nat. Med..

[24] Cook T.M., Roberts J.V. (2021). Impact of vaccination by priority group on UK deaths, hospital admissions and intensive care admissions from COVID-19. Anaesthesia.

[25] Feinstein M.M., Niforatos J.D., Hyun I., Cunningham T.V., Reynolds A., Brodie D., Levine A. (2020). Considerations for ventilator triage during the COVID-19 pandemic. Lancet Respir. Med..

[26] Angelis A., Alonso C.S., Kyriopoulos I., Mossialos E. (2022). Funding Sources of Therapeutic and Vaccine Clinical Trials for COVID-19 vs. Non–COVID-19 Indications, 2020–2021. JAMA Netw. Open.

[27] Stokel-Walker C. (2021). COVID-19: The countries that have mandatory vaccination for health workers. BMJ.

[28] Onakpoya I.J., Heneghan C.J., Aronson J.K. (2016). Worldwide withdrawal of medicinal products because of adverse drug reactions: A systematic review and analysis. Crit. Rev. Toxicol..

[29] Herxheimer A. (2012). Pharmacovigilance on the turn? Adverse reactions methods in 2012. Br. J. Gen. Pract..

[30] Brown J.P., Wing K., Evans S.J., Bhaskaran K., Smeeth L., Douglas I.J. (2019). Use of real-world evidence in postmarketing medicines regulation in the European Union: A systematic assessment of European Medicines Agency referrals 2013–2017. BMJ Open.

[31] Downing N.S., Shah N.D., Aminawung J.A., Pease A.M., Zeitoun J.D., Krumholz H.M., Ross J.S. (2017). Postmarket Safety Events Among Novel Therapeutics Approved by the US Food and Drug Administration Between 2001 and 2010. JAMA.

[32] Savage L.J. (1954). The Foundations of Statistics.

[33] Buchak L. (2014). Risk and Tradeoffs. Erkenntnis.

[34] Hall R.E., Jones C.I., Klenow P.J. (2022). Trading Off Consumption and COVID-19 Deaths. Q. Rev. Fed. Reserve Bank Minneap..

[35] Reiss J., Elliott K.C., Steel D. (2017). Meanwhile, Why Not Biomedical Capitalism?. Current Controversies in Values and Science.

[36] Flanigan J. (2017). Pharmaceutical Freedom: Why Patients Have a Right to Self Medicate.

[37] Fraile Navarro D., Tempini N., Teira D. (2021). The trade-off between impartiality and freedom in the 21st Century Cures Act. Philos. Med..

[38] Carrieri D., Peccatori F.A., Boniolo G. (2018). The ethical plausibility of the ‘Right To Try’ laws. Crit. Rev. Oncol..

[39] Jamrozik E., Selgelid M.J. (2020). COVID-19 human challenge studies: Ethical issues. Lancet Infect. Dis..

[40] World Health Organization Advisory Group on Human Challenge Studies (2020). Feasibility, Potential Value and Limitations of Establishing a Closely Monitored Challenge Model of Experimental COVID-19 Infection and Illness in Health Young Adult Volunteers.

[41] Archibald K. (2018). Animal Research Is an Ethical Issue for Humans as Well as for Animals. J. Anim. Ethics.

[42] Clark J.M. (2018). The 3Rs in research: A contemporary approach to replacement, reduction and refinement. Br. J. Nutr..

[43] Buckner J.H., Chowell G., Springborn M.R. (2021). Dynamic prioritization of COVID-19 vaccines when social distancing is limited for essential workers. Proc. Natl. Acad. Sci. USA.

[44] Wang J., Tong Y., Li D., Li J., Li Y. (2021). The Impact of Age Difference on the Efficacy and Safety of COVID-19 Vaccines: A Systematic Review and Meta-Analysis. Front. Immunol..

[45] Su Z., McDonnell D., Li X., Bennett B., Šegalo S., Abbas J., Cheshmehzangi A., Xiang Y.T. (2021). COVID-19 Vaccine Donations–Vaccine Empathy or Vaccine Diplomacy? A Narrative Literature Review. Vaccines.

[46] Ndong I.C., Okyere D., Enos J.Y., Mensah B.A., Nyarko A., Abuaku B., Amambua-Ngwa A., Merle C.S.C., Koram K.A., Ahorlu C.S. (2019). Prevalence of asymptomatic malaria parasitaemia following mass testing and treatment in Pakro sub-district of Ghana. BMC Public Health.

[47] Agyeman A.A., Ofori-Asenso R. (2016). Prevalence of HIV and hepatitis B coinfection in Ghana: A systematic review and meta-analysis. AIDS Res. Ther..

[48] Johnson G.A., Vindrola-Padros C. (2017). Rapid qualitative research methods during complex health emergencies: A systematic review of the literature. Soc. Sci. Med..

[49] Andreoletti M., Teira D. (2019). Rules versus Standards: What Are the Costs of Epistemic Norms in Drug Regulation?. Sci. Technol. Hum. Values.

[50] Osimani B. (2013). Until RCT proven? On the asymmetry of evidence requirements for risk assessment. J. Eval. Clin. Pract..

[51] Osimani B. (2013). The precautionary principle in the pharmaceutical domain: A philosophical enquiry into probabilistic reasoning and risk aversion. Health Risk Soc..

[52] Driver J., Zalta E.N., Nodelman U. (2022). The History of Utilitarianism. The Stanford Encyclopedia of Philosophy.

[53] Kavanagh K.T., Pontus C., Pare J., Cormier L.E. (2021). COVID-19 lessons learned: A global perspective. Antimicrob. Resist. Infect. Control.

[54] Nutbeam D. (2020). COVID-19: Lessons in risk communication and public trust. Public Health Res. Pract..

[55] Lancaster K., Rhodes T., Rosengarten M. (2020). Making evidence and policy in public health emergencies: Lessons from COVID-19 for adaptive evidence-making and intervention. Evid. Policy.

[56] Rubin R. (2020). Challenge Trials-Could Deliberate Coronavirus Exposure Hasten Vaccine Development?. JAMA.

[57] Pearson H. (2021). How COVID broke the evidence pipeline. Nature.

[58] Vandenbroucke J.P., Psaty B.M. (2008). Benefits and risks of drug treatments: How to combine the best evidence on benefits with the best data about adverse effects. JAMA.

[59] Beasley C.M., Dellva M.A., Tamura R.N., Morgenstern H., Glazer W.M., Ferguson K., Tollefson G.D. (1999). Randomised double-blind comparison of the incidence of tardive dyskinesia in patients with schizophrenia during long-term treatment with olanzapine or haloperidol. Br. J. Psychiatry.

[60] Duijnhoven R.G., Straus S.M.J.M., Raine J.M., de Boer A., Hoes A.W., Bruin M.L.D. (2013). Number of Patients Studied Prior to Approval of New Medicines: A Database Analysis. PLoS Med..

[61] Egger M., Schneider M., Smith G.D. (1998). Spurious precision? Meta-analysis of observational studies. BMJ.

[62] Lundh A., Lexchin J., Mintzes B., Schroll J.B., Bero L. (2017). Industry sponsorship and research outcome. Cochrane Database Syst. Rev..

[63] Ioannidis J.P. (2016). Evidence-based medicine has been hijacked: A report to David Sackett. J. Clin. Epidemiol..

[64] Etminan M., Brophy J.M., Collins G., Nazemipour M., Mansournia M.A. (2021). To Adjust or Not to Adjust: The Role of Different Covariates in Cardiovascular Observational Studies. Am. Heart J..

[65] European Medicines Agency Guideline on Adjustment for Baseline Covariates in Clinical Trials. https://www.ema.europa.eu/en/documents/scientific-guideline/guideline-adjustment-baseline-covariates-clinical-trials_en.pdf.

[66] Bovens L., Hartmann S. (2002). Bayesian Networks and the Problem of Unreliable Instruments. Philos. Sci..

[67] Landes J. (2021). The Variety of Evidence Thesis and its Independence of Degrees of Independence. Synthese.

[68] Osimani B., Landes J. (2021). Varieties of Error and Varieties of Evidence in Scientific Inference. Br. J. Philos. Sci..

[69] Abelson S.S. (2022). Variety of evidence in multimessenger astronomy. Stud. Hist. Philos. Sci..

[70] Landes J. (2020). Variety of evidence and the elimination of hypotheses. Eur. J. Philos. Sci..

[71] Dawid P. (2017). On individual risk. Synthese.

[72] Hájek A. (2007). The reference class problem is your problem too. Synthese.

[73] Bartha P., Zalta E.N. (2016). Analogy and Analogical Reasoning. The Stanford Encyclopedia of Philosophy.

[74] Khosrowi D. (2021). What is (successful) extrapolation?. J. Econ. Methodol..

[75] Benet L.Z., Sodhi J.K. (2021). Can In Vitro–In Vivo Extrapolation Be Successful? Recognizing the Incorrect Clearance Assumptions. Clin. Pharmacol. Ther..

[76] Steel D. (2010). A New Approach to Argument by Analogy: Extrapolation and Chain Graphs. Philos. Sci..

[77] Bareinboim E., Pearl J. (2013). A General Algorithm for Deciding Transportability of Experimental Results. J. Causal Inference.

[78] Wilde M., Parkkinen V.P. (2019). Extrapolation and the Russo-Williamson thesis. Synthese.

[79] Dutilh G., Sarafoglou A., Wagenmakers E.J. (2021). Flexible yet fair: Blinding analyses in experimental psychology. Synthese.

[80] Ioannidis J.P.A. (2005). Why Most Published Research Findings Are False. PLoS Med..

[81] Dardashti R., Hartmann S., Thebault K.P.Y., Winsberg E. (2019). Hawking Radiation and Analogue Experiments: A Bayesian Analysis. Stud. Hist. Philos. Mod. Phys..

[82] Jukola S. (2019). On the evidentiary standards for nutrition advice. Stud. Hist. Philos. Sci. Part C Stud. Hist. Philos. Biol. Biomed. Sci..

[83] World Health Organization (2020). Healthy Diet. https://www.who.int/news-room/fact-sheets/detail/healthy-diet.

[84] Reiss J. (2013). Philosophy of Economics.

[85] Giannuzzi V., Conte R., Landi A., Ottomano S.A., Bonifazi D., Baiardi P., Bonifazi F., Ceci A. (2017). Orphan medicinal products in Europe and United States to cover needs of patients with rare diseases: An increased common effort is to be foreseen. Orphanet J. Rare Dis..

[86] Daniali H., Flaten M.A. (2021). Experiencing COVID-19 symptoms without the disease: The role of nocebo in reporting of symptoms. Scand. J. Public Health.

[87] Siguan A.A. (2022). Conspiracies and the nocebo effect during the COVID-19 pandemic. J. Public Health.

[88] World Health Organization (2021). Solidarity Trial PLUS: Protocol. https://www.who.int/publications/m/item/solidarity-trial-plus-protocol.

[89] Leonelli S. (2021). Data Science in Times of Pan(dem)ic. Harv. Data Sci. Rev..

[90] Jukola S., Canali S. (2021). On evidence fiascos and judgments in COVID-19 policy. Hist. Philos. Life Sci..

[91] European Medicines Agency (2018). Accelerated Assessment. https://www.ema.europa.eu/en/human-regulatory/marketing-authorisation/accelerated-assessment.

[92] Food and Drug Administration (2023). Emergency Use Authorization. https://www.fda.gov/emergency-preparedness-and-response/mcm-legal-regulatory-and-policy-framework/emergency-use-authorization.

[93] Singh J.A., Upshur R.E.G. (2021). The granting of emergency use designation to COVID-19 candidate vaccines: Implications for COVID-19 vaccine trials. Lancet Infect. Dis..

[94] Raphael M.J., Gyawali B., Booth C.M. (2020). Real-world evidence and regulatory drug approval. Nat. Rev. Clin. Oncol..

[95] De Pretis F., van Gils M., Forsberg M.M. (2022). A smart hospital-driven approach to precision pharmacovigilance. Trends Pharmacol. Sci..

[96] Mebius A., Kennedy A.G., Howick J. (2016). Research gaps in the philosophy of evidence-based medicine. Philos. Compass.

[97] Stegenga J. (2014). Herding QATs: Quality Assessment Tools for Evidence in Medicine. Classification, Disease and Evidence.

[98] Ioannidis J.P. (2016). The Mass Production of Redundant, Misleading, and Conflicted Systematic Reviews and Meta-analyses. Milbank Q..

[99] Tricco A.C., Soobiah C., Antony J., Cogo E., MacDonald H., Lillie E., Tran J., D’Souza J., Hui W., Perrier L. (2016). A scoping review identifies multiple emerging knowledge synthesis methods, but few studies operationalize the method. J. Clin. Epidemiol..

[100] Greenhalgh T., Wong G., Westhorp G., Pawson R. (2011). Protocol – realist and meta-narrative evidence synthesis: Evolving Standards (RAMESES). BMC Med Res. Methodol..

[101] van den Berg T., Heymans M.W., Leone S.S., Vergouw D., Hayden J.A., Verhagen A.P., de Vet H.C. (2013). Overview of data-synthesis in systematic reviews of studies on outcome prediction models. BMC Med Res. Methodol..

[102] Shinkins B., Yang Y., Abel L., Fanshawe T.R. (2017). Evidence synthesis to inform model-based cost-effectiveness evaluations of diagnostic tests: A methodological review of health technology assessments. BMC Med Res. Methodol..

[103] Kent S., Salcher-Konrad M., Boccia S., Bouvy J.C., de Waure C., Espin J., Facey K., Nguyen M., Rejon-Parrilla J.C., Jonsson P. (2021). The use of nonrandomized evidence to estimate treatment effects in health technology assessment. J. Comp. Eff. Res..

[104] Verde P.E., Ohmann C. (2014). Combining randomized and non-randomized evidence in clinical research: A review of methods and applications. Res. Synth. Methods.

[105] Fletcher S.C., Landes J., Poellinger R. (2019). Evidence Amalgamation in the Sciences: An Introduction. Synthese.

[106] Hansson S.O. (2022). Can Uncertainty Be Quantified?. Perspect. Sci..

[107] Howson C., Urbach P. (2006). Scientific Reasoning.

[108] Sprenger J., Hartmann S. (2019). Bayesian Philosophy of Science.

[109] De Pretis F., Jukola S., Landes J. (2022). E-Synthesis for Carcinogenicity Assessments: A Case Study of Processed Meat. J. Eval. Clin. Pract..

[110] Spiegelhalter D., Myles J., Jones D., Abrams K. (2000). Bayesian methods in health technology assessment: A review. Health Technol. Assess..

[111] Blackwell D., Dubins L. (1962). Merging of Opinions with Increasing Information. Ann. Math. Stat..

[112] Landes J., Osimani B., Poellinger R. (2018). Epistemology of Causal Inference in Pharmacology. Eur. J. Philos. Sci..

[113] De Pretis F., Landes J., Osimani B. (2019). E-Synthesis: A Bayesian Framework for Causal Assessment in Pharmacosurveillance. Front. Pharmacol..

[114] Abdin A.Y., Auker-Howlett D.J., Landes J., Mulla G., Jacob C., Osimani B. (2019). Reviewing the Mechanistic Evidence Assessors E-Synthesis and EBM+: A Case Study of Amoxicillin and Drug Reaction with Eosinophilia and Systemic Symptoms (DRESS). Curr. Pharm. Des..

[115] De Pretis F., Landes J. (2021). EA^3^: A softmax algorithm for evidence appraisal aggregation. PLoS ONE.

[116] Landes J., Auker-Howlett D.J. (2023). Drug (Dis-)Approval in the Real World.

[117] de Finetti B. (1972). Probability, Induction and Statistics.

[118] Lindley D.V. (2000). The Philosophy of Statistics. J. R. Stat. Soc. Ser. D (The Stat.).

[119] Sprenger J. (2018). The objectivity of Subjective Bayesianism. Eur. J. Philos. Sci..

[120] Radzvilas M., Peden W., De Pretis F. (2021). A Battle in the Statistics Wars: A simulation-based comparison of Bayesian, Frequentist and Williamsonian methodologies. Synthese.

[121] Williamson J. (2010). Defence of Objective Bayesianism.

[122] Landes J., Williamson J. (2013). Objective Bayesianism and the maximum entropy principle. Entropy.

[123] Goodman N. (1946). A Query on Confirmation. J. Philos..

[124] Shafer G., Vovk V. (2001). Probability and Finance.

[125] (2014). Miranda, Enrique and Cooman, Gert de Lower Previsions.

[126] Bradley S., Zalta E.N. (2015). Imprecise Probabilities. Stanford Encyclopedia of Philosophy.

[127] Troffaes M.C. (2007). Decision making under uncertainty using imprecise probabilities. Int. J. Approx. Reason..

[128] Doyle J., Thomason R.H. (1999). Background to Qualitative Decision Theory. AI Mag..

[129] Spohn W. (2012). The Laws of Belief. Ranking Theory and Its Philosophical Applications.

[130] Mayo D.G. (2018). Statistical Inference as Severe Testing: How to Get Beyond the Statistics Wars.

[131] Barberà S., Bossert W., Pattanaik P.K., Barbera S., Hammond P., Seidl C. (2002). Ranking Sets of Objects. Handbook of Utility Theory.

[132] Bossert W., Pattanaik P.K., Xu Y. (2000). Choice under complete uncertainty: Axiomatic characterizations of some decision rules. Econ. Theory.

[133] Ahn D.S. (2008). Ambiguity Without a State Space. Rev. Econ. Stud..

[134] Tillman R.E., Eberhardt F. (2014). Learning causal structure from multiple datasets with similar variable sets. Behaviormetrika.

[135] Lee C.Y., Chen Y.P.P. (2019). Machine learning on adverse drug reactions for pharmacovigilance. Drug Discov. Today.

[136] Mockute R., Desai S., Perera S., Assuncao B., Danysz K., Tetarenko N., Gaddam D., Abatemarco D., Widdowson M., Beauchamp S. (2019). Artificial Intelligence Within Pharmacovigilance: A Means to Identify Cognitive Services and the Framework for Their Validation. Pharm. Med..

[137] Price J. (2016). What Can Big Data Offer the Pharmacovigilance of Orphan Drugs?. Clin. Ther..

[138] Sardella M., Belcher G. (2018). Pharmacovigilance of medicines for rare and ultrarare diseases. Ther. Adv. Drug Saf..

[139] Landes J., Williamson J. (2022). Objective Bayesian Nets for Integrating Consistent Datasets. J. Artif. Intell. Res..

[140] Ras G., Xie N., Gerven M.V., Doran D. (2022). Explainable Deep Learning: A Field Guide for the Uninitiated. J. Artif. Intell. Res..

[141] De Pretis F., Landes J., Peden W.J. (2021). Artificial Intelligence Methods For a Bayesian Epistemology-Powered Evidence Evaluation. J. Eval. Clin. Pract..

[142] Parker W.S. (2022). Evidence and Knowledge from Computer Simulation. Erkenntnis.

[143] Boge F.J. (2022). Why trust a simulation? Models, parameters, and robustness in simulation-infected experiments. Br. J. Philos. Sci..

[144] Mayo-Wilson C. (2014). The Limits of Piecemeal Causal Inference. Br. J. Philos. Sci..

[145] Osimani B., Bertolaso M., Poellinger R., Frontoni E. (2019). Real and Virtual Clinical Trials: A Formal Analysis. Topoi.

[146] Parkkinen V.P., Wallmann C., Wilde M., Clarke B., Illari P., Kelly M.P., Norell C., Russo F., Shaw B., Williamson J. (2018). Evaluating Evidence of Mechanisms in Medicine: Principles and Procedures.

[147] Greenhalgh T., Fisman D., Cane D.J., Oliver M., Macintyre C.R. (2022). Adapt or die: How the pandemic made the shift from EBM to EBM+ more urgent. BMJ Evid.-Based Med..

[148] Sandifer P., Knapp L., Lichtveld M., Manley R., Abramson D., Caffey R., Cochran D., Collier T., Ebi K., Engel L. (2020). Framework for a Community Health Observing System for the Gulf of Mexico Region: Preparing for Future Disasters. Front. Public Health.

[149] Adu-Gyamfi B., Shaw R. (2022). Risk Awareness and Impediments to Disaster Preparedness of Foreign Residents in the Tokyo Metropolitan Area, Japan. Int. J. Environ. Res. Public Health.

[150] Haque A., Haider D., Rahman M.S., Kabir L., Lejano R.P. (2022). Building Resilience from the Grassroots: The Cyclone Preparedness Programme at 50. Int. J. Environ. Res. Public Health.

[151] Thorstad D. (2022). General-Purpose Institutional Decision-Making Heuristics: The Case of Decision-Making under Deep Uncertainty. Br. J. Philos. Sci..

[152] Park S.W. (2022). A reflection on health and disease amid COVID-19 pandemic. J. Eval. Clin. Pract..

[153] Chou F.L., Abramson D., DiMaggio C., Hoven C.W., Susser E., Andrews H.F., Chihuri S., Lang B.H., Ryan M., Herman D. (2021). Factors Related to Self-Reported Distress Experienced by Physicians During Their First COVID-19 Triage Decisions. Disaster Med. Public Health Prep..

[154] Arcaya M., Raker E.J., Waters M.C. (2020). The Social Consequences of Disasters: Individual and Community Change. Annu. Rev. Sociol..

